# CSF1R-dependent CD169-positive macrophages locally constrain melanoma growth in the skin

**DOI:** 10.1084/jem.20252239

**Published:** 2026-05-21

**Authors:** Yuki Honda Keith, Emily Duchini, Xufeng Lin, Wunna Kyaw, Felix G.P. Weninger, Rama Dhenni, Aiden Josiah Telfser, Abigail K. Grootveld, Deborah Barkauskas, Angela Fontaine-Titley, Shweta Tikoo, Rohit Jain, Wolfgang Weninger, John W. Frew, Elissa K. Deenick, Robert Brink, Linda K. Martin, Tatyana Chtanova, Leonard D. Goldstein, Richard A. Scolyer, Georgina V. Long, Umaimainthan Palendira, Tri Giang Phan

**Affiliations:** 1 Precision Immunology Program, Garvan Institute of Medical Research, Sydney, Australia; 2 Faculty of Medicine, St Vincent’s Clinical School, University of New South Wales, Sydney, Australia; 3 Faculty of Medicine and Health, The University of Sydney, Sydney, Australia; 4 Charles Perkins Centre, The University of Sydney, Sydney, Australia; 5 Computational Biology Group, Data Science Platform, Garvan Institute of Medical Research, Sydney, Australia; 6 Faculty of Medicine, The School of Biomedical Sciences, University of New South Wales, Sydney, Australia; 7 Faculty of Science, School of Biotechnology and Biomolecular Sciences, University of New South Wales, Sydney, Australia; 8 ACRF INCITe Centre for Intravital Imaging, Garvan Imaging Platform, Garvan Institute of Medical Research and the Kinghorn Cancer Centre, Sydney, Australia; 9 Department of Dermatology, The Medical University of Vienna, Vienna, Austria; 10 School of Clinical Medicine, University of New South Wales, Sydney, Australia; 11 Immunovirology and Pathogenesis Program, Kirby Institute, University of New South Wales, Sydney, Australia; 12 Immune Biotherapies Program, Garvan Institute of Medical Research, Sydney, Australia; 13 Melanoma Institute Australia, The University of Sydney, Sydney, Australia; 14 Royal Prince Alfred Hospital, Sydney, Australia; 15 NSW Health Pathology, Sydney, Australia; 16 Mater Hospital, North Sydney, Australia; 17 Royal North Shore Hospital, St Leonards, Australia

## Abstract

Macrophages in the skin reside in multiple distinct layers and perform various functions. Here, we show that CD169^+^ macrophages reside in the hypodermis and comprise the major skin myeloid cell population in the steady state. In a syngeneic melanoma model, CD169^+^ macrophages encapsulate growing melanomas and directly suppress their growth. CSF1R blockade depleted CD169^+^ macrophages in tumors and resulted in unrestrained growth. This local containment of tumor growth in the skin was independent of CD169^+^ subcapsular sinus macrophages in the tumor-draining lymph node and did not require B or T cells. Intravital imaging revealed engulfment and ingestion of live tumor cells by CD169^+^ macrophages. This phagocytosis did not require the phosphatidylserine receptor MERTK. CD169^+^ macrophages are also enriched in the hypodermis in skin biopsies from healthy human skin and melanoma. These data identify tissue-resident CD169^+^ macrophages as a potential cellular target to achieve innate immune containment and reinforce adaptive immune control of tumors.

## Teaser

Tissue-resident CSF1R-dependent CD169^+^ macrophages localize deep in the skin. They surround growing tumors and directly suppress their growth independent of T and B cells. CD169^+^ skin macrophages ingest live tumors, and this phagocytosis is independent of the phosphatidylserine receptor MERTK.

## Introduction

The skin is the largest organ in the body and is constantly exposed to various external threats, including toxins, irritants, and pathogens. It is organized into three microanatomical layers, the epidermis, dermis, and hypodermis, that contain different cell types and structures that also serve different immune functions (Kabashima et al., 2019). In the steady state, tissue-resident macrophages can be found in the epidermis, where they form a strategic network of sentinels to monitor the integrity of the skin barrier and surveil for pathogens, and in the dermis, where they play important roles in tissue homeostasis, wound healing, chemical irritation, pathogen surveillance, allergic inflammation, and autoimmune disease (Malissen et al., 2014). These diverse roles suggest skin macrophages may comprise a heterogeneous group of cells with different subpopulations adapted to perform various functions in other locations (Mass et al., 2023), such as perineural (Kolter et al., 2019) and perivascular macrophages (Abtin et al., 2014; Barreiro et al., 2016). However, the identity and role of skin macrophage subpopulations in anti-tumor immunity are yet to be fully elucidated.

Malignant melanoma is the most aggressive skin cancer arising from melanocytes in the skin. The fate of malignant cancers such as melanoma can be controlled by the tumor immune microenvironment (TIME), where cancer cells closely interact with both immune and nonimmune cells (Binnewies et al., 2018), and the extra-TIME (EXTRA-TIME), particularly in the tumor-draining LN (tdLN), where anti-tumor adaptive immune responses are staged (Phan et al., 2024). Tumor-associated macrophages (TAMs) are among the most abundant and functionally heterogeneous cells in the TIME (DeNardo and Ruffell, 2019). In clinical studies, tumor-promoting TAMs are associated with poor prognosis in patients with melanoma (Torisu et al., 2000; Jensen et al., 2009). In contrast, in the preclinical B16-F10 melanoma mouse model, tumor-infiltrating CD206^+^ macrophages have been shown to cross-present tumor antigens to activate cytotoxic CD8^+^ T cells (Modak et al., 2022), suggesting they may also have an anti-tumor role. Not surprisingly, the targeting of macrophages, for example, with colony-stimulating factor 1 receptor (CSF1R) inhibitors or blocking monoclonal antibodies (mAbs), has generated conflicting results in preclinical mouse models and human clinical trials (Habib et al., 2024). This may have arisen due to the heterogeneity and plasticity of macrophages in the TIME.

The role of macrophages in the EXTRA-TIME is also controversial. Live and apoptotic tumor cells and tumor-derived extracellular vesicles may drain from the skin in the afferent lymphatics to the tdLN, where they initially come into contact with CD169^+^ subcapsular sinus macrophages (SSM) (Moran et al., 2019). These CD169^+^ macrophages play an essential role in promoting adaptive humoral immunity by presenting captured antigens to naive B cells (Carrasco and Batista, 2007; Junt et al., 2007; Phan et al., 2007), memory B cells (Moran et al., 2018; Dhenni et al., 2025) and memory T follicular helper cells (Suan et al., 2015). Clinically, the higher density of CD169^+^ macrophages in tdLN is correlated with better prognosis in patients with various cancers (Komohara et al., 2017). CD169^+^ macrophages have been shown to cross-present antigens derived from dead tumor cells to cytotoxic CD8^+^ T cells (Asano et al., 2011). They have also been shown to suppress melanoma growth, possibly by restricting interaction between B cells and tumor-derived extracellular vesicles (Pucci et al., 2016). However, these animal studies either used CD169-DTR transgenic mice or liposomal clodronate to systemically deplete CD169^+^ macrophages in the whole animal. Therefore, it is not clear if the results from these experiments are due to the specific depletion of CD169^+^ macrophages in the tdLN or in the tumor itself. In this report, we describe CD169^+^ macrophages that reside near blood vessels in the hypodermis and show that they phagocytose live tumor cells to directly control the growth of syngeneic B16-F10 melanomas in the skin.

## Results and discussion

### Identification of CD169^+^ macrophages in murine skin

**Figure 1.**
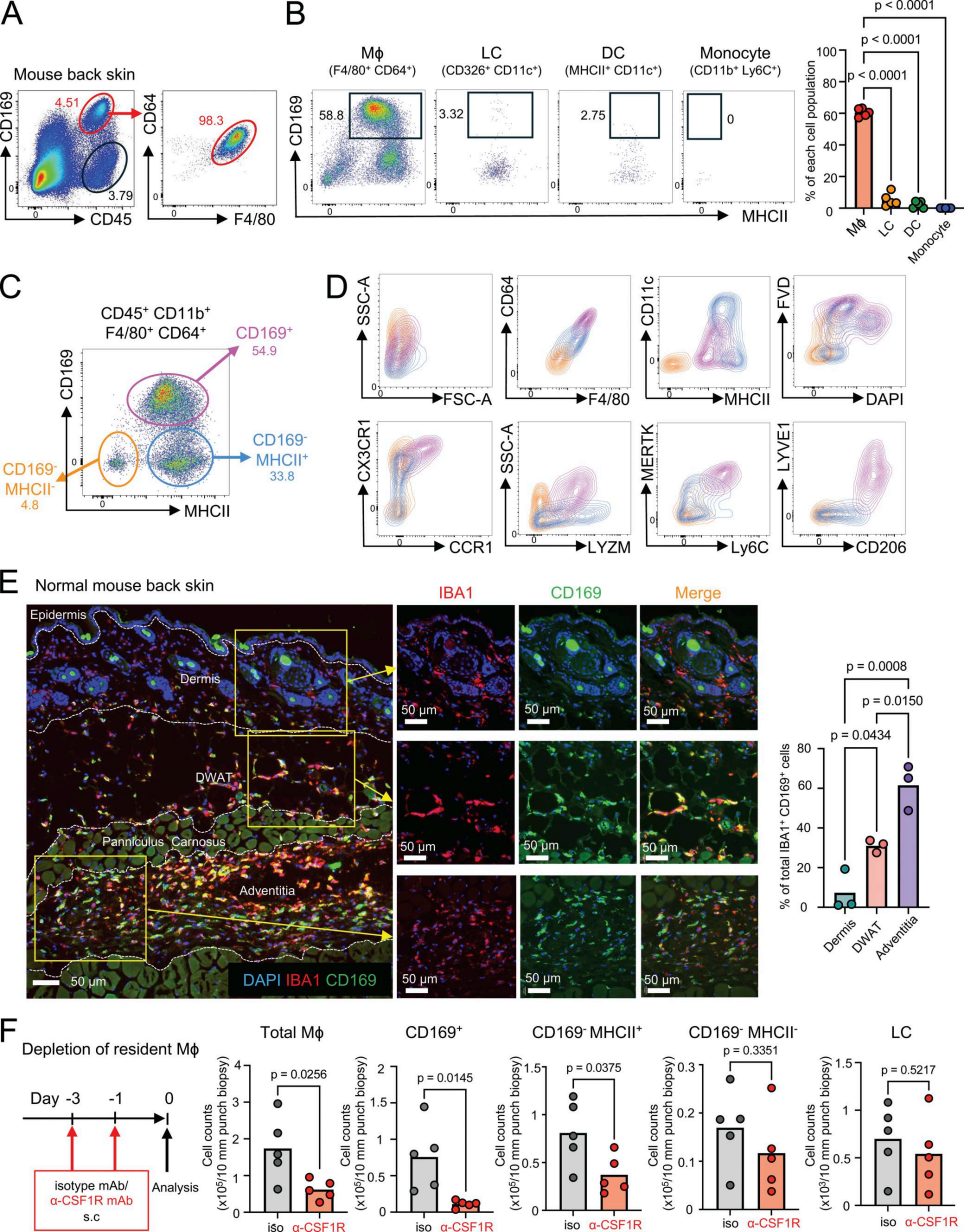
**Identification of CSF1R-dependent CD169^+^ macrophages in the normal skin. (A)** Representative flow cytometry plots with gates showing CD169^+^ macrophages in the mouse normal back skin (representative of five mice). **(B)** Representative flow cytometry plots of CD169 expression in skin macrophages (Mϕ), LCs, DCs, and monocytes (representative of five mice). The graph indicated the percentages of CD169^+^ cells in each cell population. P value was calculated using one-way ANOVA. **(C)** Three macrophage populations based on MHCII and CD169 expression. **(D)** Representative flow cytometry plots of CD64, F4/80, CD11c, MHCII, CCR1, CX3CR1, LYZM, MERTK, Ly6C, LYVE1, and CD206 and positivity of DAPI and FVD in CD169^+^ Mϕ (magenta), CD169^−^ MHCII^+^ Mϕ (blue), and CD169^−^ MHCII^−^ Mϕ (orange) (representative of at least three mice). **(E)** Immunostaining of IBA1 (red), CD169 (green), and DAPI (blue) in mouse normal back skin. The ratio of CD169^+^ cells in the dermis, DWAT, and adventitia was analyzed using Imaris (*n* = 3). Scale bar = 50 μm. P value was calculated using one-way ANOVA. **(F)** Experimental scheme for CD169^+^ macrophage depletion by anti-CSF1R mAb. 200 μg isotype mAb or anti-CSF1R mAb was subcutaneously injected into the mouse back skin on days −3 and −1, and then each skin macrophage population and LC were analyzed on day 0 using flow cytometry (*n* = 5 for each group, data represent two independent experiments). Each circle represents one mouse. P value was calculated using Student’s *t* test.

**Figure S1.**
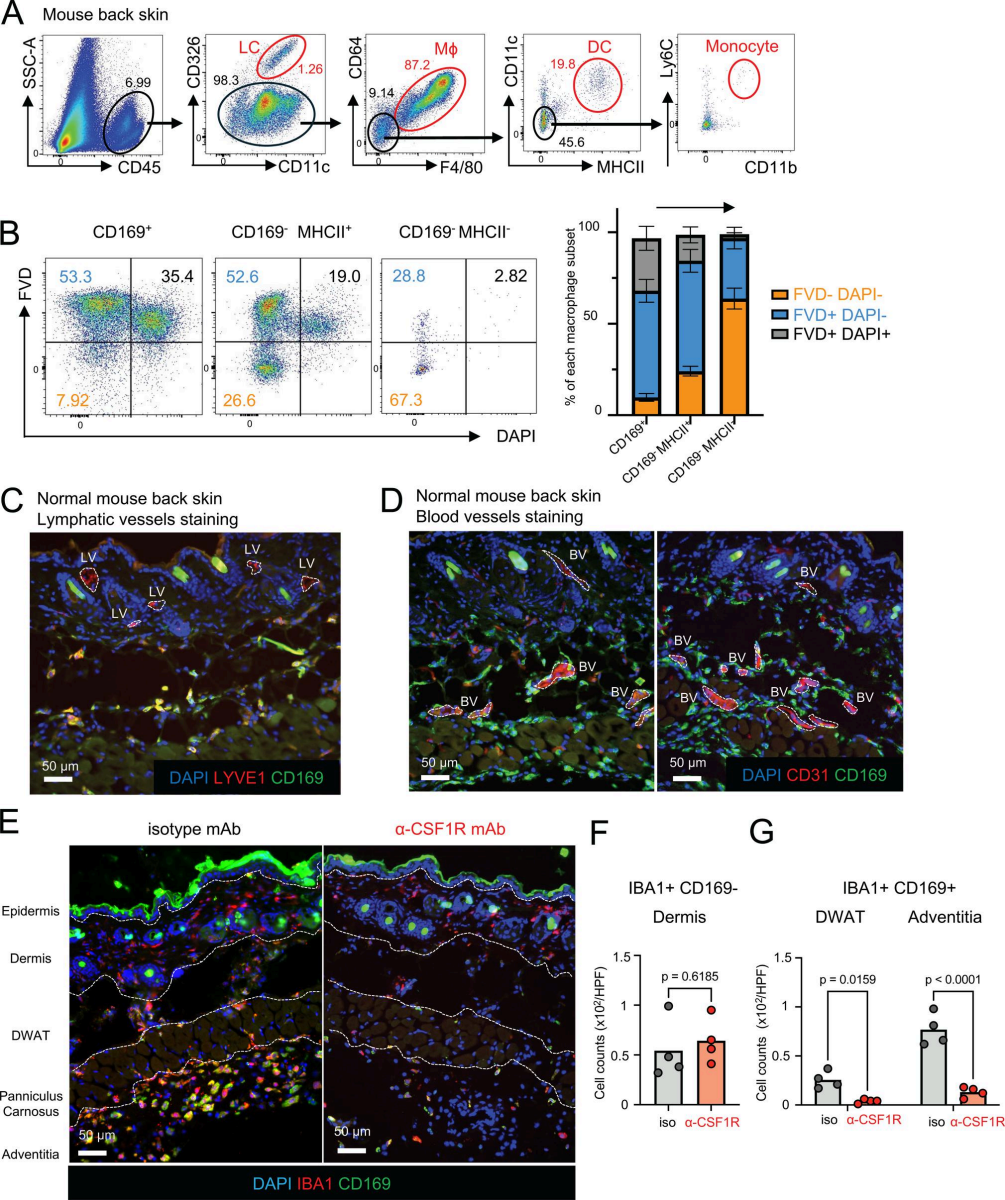
**Skin CD169+ macrophages are CSF1R-dependent perivascular macrophages** **. (A)** Gating strategy to identify skin LCs, macrophages (Mϕ), DCs, and monocytes (representative of five mice). **(B)** The ratio of FVD^–^ DAPI^–^ (orange), FVD^+^ DAPI^–^ (aqua), and FVD^+^ DAPI^+^ (black) in CD169^+^ Mϕ, CD169^–^ MHCII^+^ Mϕ, and CD169^–^ MHCII^–^ Mϕ (*n* = 5, data are shown with mean ± SD, data are representative of two independent experiments). **(C)** Immunostaining of CD169 (green), CD31 (red), and DAPI (blue) in normal mouse back skin. BV, blood vessels. **(D)** Immunostaining of CD169 (green), LYVE1 (red), and DAPI (blue) in normal mouse back skin. LV, lymphatic vessels. **(E)** Immunostaining of IBA1 (red), CD169 (green), and DAPI (blue) in mouse back skin after two treatments of subcutaneous injection of either isotype mAb or anti-CSF1R mAb. **(F)** The number of IBA1^+^ CD169^−^ cells in the dermis per HPF was analyzed using Imaris (×20) (*n* = 4 for each group). P value was calculated using Student’s *t* test. **(G)** The number of IBA1^+^ CD169^+^ cells in the DWAT, and adventitia per HPF was analyzed using Imaris (×20) (*n* = 4 for each group). Each circle represents one mouse. P value was calculated using Student’s *t* test.

We used a panel of cell surface markers to resolve the different macrophage subpopulations in the mouse full-thickness back skin by flow cytometric analysis. Single-cell suspension was prepared for flow cytometric analysis using a collagenase digestion protocol that was optimized for cell recovery and detection of cell surface molecules. The C-type lectin CD169, also known as sialoadhesin and encoded by the *Siglec1* gene, was shown to be expressed on a subpopulation of dermal macrophages (Dupasquier et al., 2004; Park et al., 2022; Li et al., 2024). Our analysis showed that CD169^+^ macrophages expressing F4/80 and CD64 comprised the major population of CD45^+^ immune cells in the steady-state skin (Fig. 1 A). Dermal/hypodermal macrophages could be distinguished from Langerhans cells (LC), dendritic cells (DC), and monocytes by the differential expression levels of EpCAM (CD326), CD11c, F4/80, CD64, the major histocompatibility class II antigen (MHCII), Ly6C, and CD11b (Fig. S1 A). This revealed that tissue resident macrophages were the major myeloid cell population that expressed CD169 in the skin (Fig. 1 B). Skin macrophages could be further separated based on the expression of CD169 and MHCII into CD169^+^, CD169^−^MHCII^+^ and CD169^−^MHCII^−^ subpopulations (Fig. 1 C). CD169^+^ macrophages were all MHCII^+^. They exhibited high side scatter and expressed higher levels of F4/80, CD64, CX3CR1, CCR1, Mer tyrosine kinase (MERTK), lysozyme M (LYZM), Ly6C, lymphatic vessel endothelial hyaluronan receptor 1 (LYVE1), and CD206 compared with the CD169-negative macrophages (Fig. 1 D). The high expression of CD206 suggests that CD169^+^ macrophages may be responsible for cross-presenting tumor antigens to CD8^+^ T cells (Modak et al., 2022). Interestingly, the majority of CD169^+^ macrophages stained with the fixable viability dye (FVD) and may otherwise be gated out as “dead cells” during flow cytometric analysis (Fig. S1 B). This may reflect their higher phagocytic capacity due to their higher expression of the phagocytic receptors F4/80, CD64, MERTK, LYZM, and CD206.

### CD169^+^ macrophages reside in the hypodermis and are CSF1R dependent

IBA1 is a calcium-binding protein specifically expressed in microglia/macrophages that participates in membrane ruffling and phagocytosis (Sasaki et al., 2001). Immunofluorescence (IF) microscopic analysis of the normal mouse back skin revealed that CD169 stained IBA1^+^ macrophages located deep in the dermal white adipose tissue (DWAT) and adventitia, but not those in the upper dermis (Fig. 1 E). In agreement with the flow cytometric analysis (Fig. 1 D), CD169^+^ macrophages stained for LYVE1 (Fig. S1 C), a marker of perivascular macrophages (Chakarov et al., 2019). They were positioned along CD31^+^ blood vessels, but not LYVE1^+^ lymphatic vessels (Fig. S1, C and D). CSF1R signalling is critical for the survival of resident macrophages in various tissues, including the skin (Hume and MacDonald, 2012). CD169^+^ tissue resident macrophages express CSF1R (Grabert et al., 2020) and are sensitive to CSF1R blockade (Hume and MacDonald, 2012). Consistent with this, subcutaneous injection of a blocking anti-CSF1R mAb (MacDonald et al., 2010) depleted CD169^+^ and CD169^−^ MHCII^+^ macrophages locally in the skin (Fig. 1 F). Depletion was most profound in the CD169^+^ macrophages in the hypodermis (Fig. S1, E–G). There was no significant decrease in the number of CD169^−^ MHCII^−^ macrophages and LCs. CSF1R blockade has previously been shown to deplete LCs (MacDonald et al., 2010), and the discrepancy may reflect differences in sensitivity and accessibility of the anti-CSF1R mAb to LCs in the epidermis. It may also reflect differences in the dose and route of administration of the CSF1R-blocking mAb we used in comparison with the original study.

### CSF1R-dependent CD169^+^ skin macrophages constrain tumor growth

**Figure 2.**
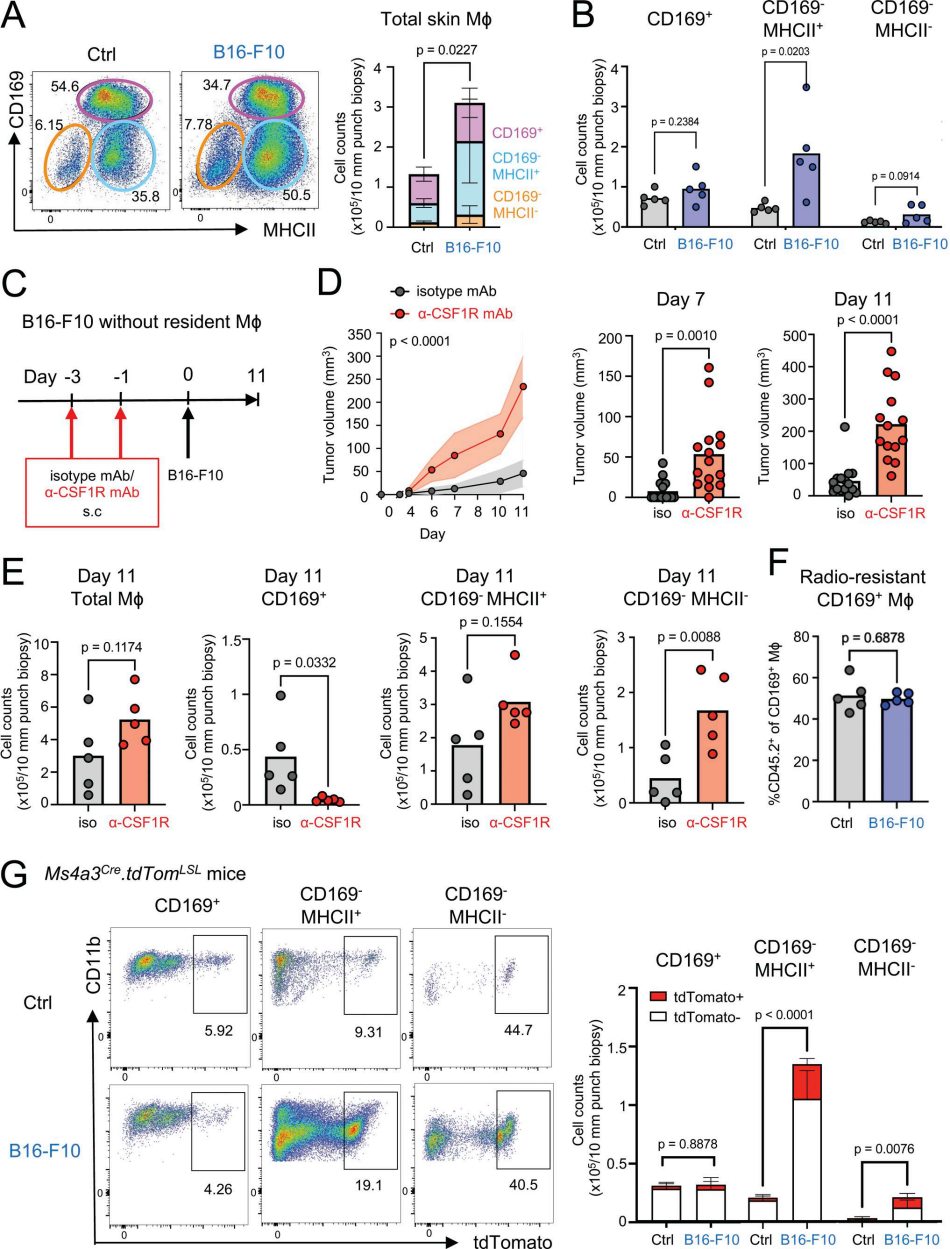
**Skin CD169^+^ macrophages suppress B16-F10 melanoma growth. (A and B)** Representative flow cytometry plots of skin Mϕ (CD45^+^ CD11b^+^ CD64^+^ F4/80^+^ cells) in control or B16-F10–bearing skin (day 11). The number of total Mϕ, including CD169^+^ Mϕ, CD169^−^ MHCII^+^ Mϕ, and CD169^−^ MHCII^−^ Mϕ in control (Ctrl) or B16-F10–bearing skin, were analyzed using flow cytometry (*n* = 5 for each group, data are shown with mean ± SD, representative of two independent experiments). P value was calculated using Student’s *t* test. **(C)** Experimental scheme for the depletion of skin-resident macrophages before B16-F10 tumor implantation. 200 μg isotype mAb or anti-CSF1R mAb was subcutaneously injected on days −3 and −1, and then 1.0 × 10^5^ B16-F10 cells were intradermally/subcutaneously injected into the mouse back skin on day 0. **(D)** The tumor volumes of B16-F10 melanoma with isotype mAb versus anti-CSF1R antibodies (*n* = 15 for each group, data are combined from three independent experiments with five mice per group. The results are expressed as the mean with a 95% confidence interval). P value was calculated using two-way ANOVA. **(E)** The numbers of skin total Mϕ, CD169^+^ Mϕ, CD169^−^ MHCII^+^ Mϕ, and CD169^–^ MHCII^–^ Mϕ in the tumor-bearing skin were analyzed using flow cytometry on day 11 under treatment with isotype mAb or anti-CSF1R mAb (*n* = 4–5 for each group, data are representative of two independent experiments). P value was calculated using Student’s *t* test. **(F)** The ratio of CD45.2^+^ cells in CD169^+^ Mϕ in normal or B16-F10–bearing skin on day 12 was analyzed by flow cytometry. 1.0 × 10^5^ B16-F10 cells were intradermally/subcutaneously injected into the mouse back skin 10–12 wk after the lethal irradiation, followed by BM cell transfer from CD45.1^+^ congenic mice (*n* = 5 for each group, data are representative of two independent experiments). P value was calculated using Student’s *t* test. **(G)** Representative flow cytometry plots of CD169^+^ Mϕ, CD169^−^ MHCII^+^ Mϕ, CD169^–^ MHCII^–^ Mϕ in control or B16-F10–bearing skin of *Ms4a3*^*cre*^.*tdTom*^*LSL*^ mice (day 9). The numbers of Tomato-positive and -negative CD169^+^ Mϕ, CD169^–^ MHCII^+^ Mϕ, and CD169^–^ MHCII^–^ Mϕ in control (Ctrl) or B16-F10–bearing skin were analyzed using flow cytometry (*n* = 4 for each group, data are shown with mean ± SD, representative of two independent experiments). Each circle represents one mouse. P value was calculated using Student’s *t* test.

We next determined the skin macrophage response to the subcutaneous implantation of syngeneic B16-F10 melanoma cells. Flow cytometric analysis of the skin 11 days later revealed total number of macrophages was significantly increased (Fig. 2 A). This was largely due to an increase in the CD169^−^MHCII^+^ macrophages, while the number of CD169^+^ macrophages did not significantly increase (Fig. 2 B). To determine the role of skin-resident macrophages in tumor growth, we injected anti-CSF1R mAb on days −3 and −1 before B16-F10 implantation (Fig. 2 C). Compared with the isotype control, there was unrestrained tumor growth, especially at day 7 and day 11 (Fig. 2 D). Flow cytometric analysis revealed that, in the presence of melanoma cells, anti-CSF1R mAb led to depletion of the CD169^+^ macrophages (Fig. 2 E). In contrast to the steady state (Fig. 1 F), there was no depletion of CD169^−^MHCII^+^ macrophages, suggesting that these may have been replenished by CSF1R-independent precursors in the presence of melanomas (Fig. 2 E). We next lethally irradiated CD45.2^+^ recipient mice and reconstituted them with bone marrow (BM) from congenic CD45.1^+^ donor mice. Flow cytometric analysis of reconstituted skin showed that ∼50% of CD169^+^ skin macrophages were derived from radio-resistant CD45.2^+^ recipient cells in both the absence and presence of melanoma (Fig. 2 F). In addition, we utilized Ms4a3Cre.tdTomLSL mice (Liu et al., 2019) to determine the contribution of Ms4a3-lineage circulating monocytes to each macrophage population in the steady state and in tumor-bearing skin. In normal skin, the majority of CD169^+^ and CD169^−^ MHCII^+^ macrophages did not contain tdTomato^+^ cells, while about half of CD169^−^ MHCII^−^ cells were tdTomato^+^. In B16-F10 melanoma skin, tdTomato^+^ cells were not increased for CD169^+^ macrophages but were increased for CD169^−^ MHCII^+^ and CD169^−^ MHCII^−^ cells (Fig. 2 G). These findings suggest that CD169^+^ macrophages were a distinct macrophage population that is locally maintained with minimal contribution of monocytes. Thus, we conclude that partially radio-resistant tissue-resident CD169^+^ macrophages are indispensable for the containment of tumor growth in the skin. However, since the anti-CSF1R mAb also increased the CD169^−^MHCII^−^ macrophage population, we cannot exclude the possible contribution of this and other off-target effects on tumor control.

### CSF1R-dependent CD169^+^ skin macrophages surround growing tumors

**Figure 3.**
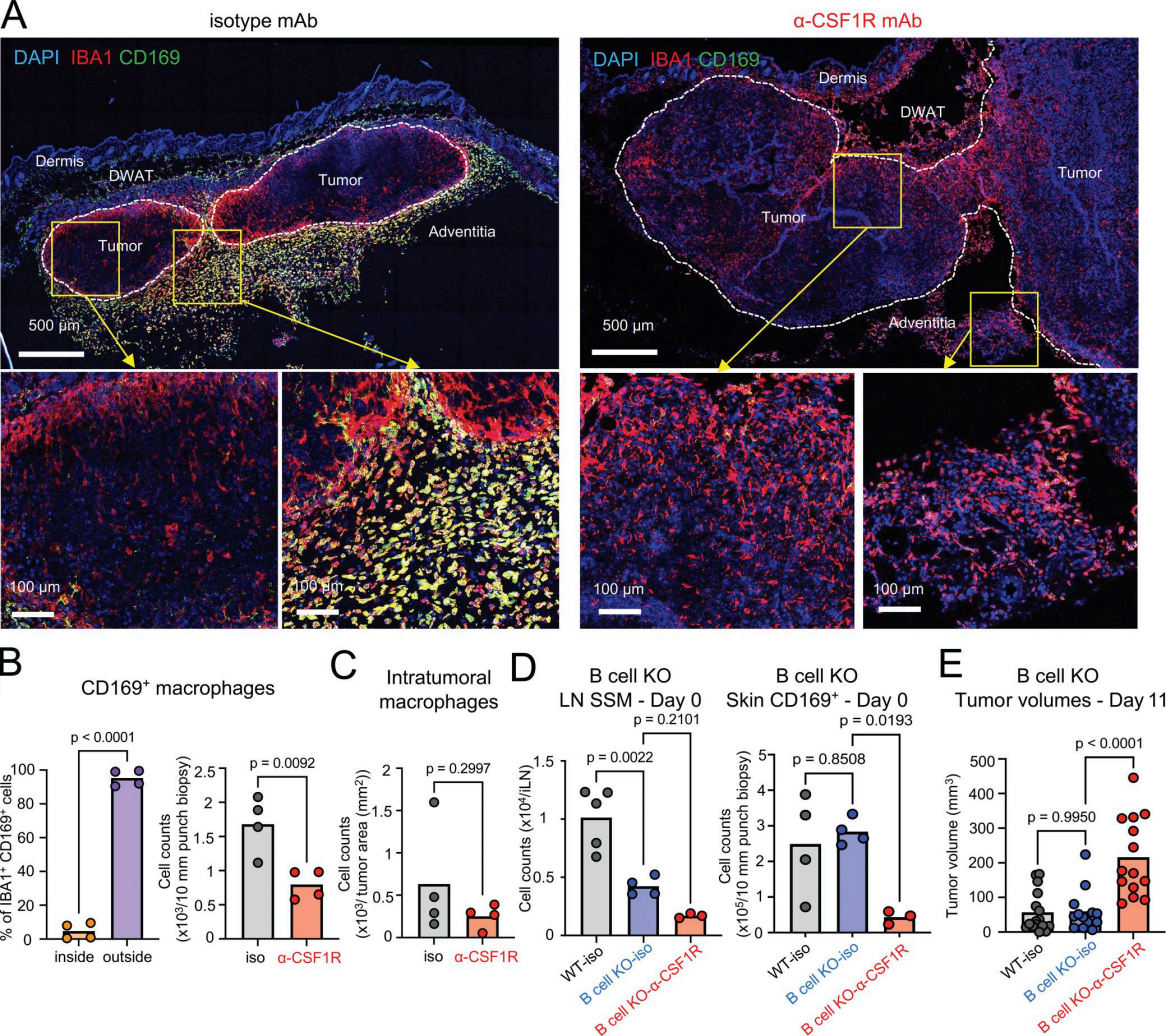
**Skin CD169^+^ macrophages surround the tumor. (A)** Immunostaining of IBA1 (red), CD169 (green), and DAPI (blue) in mouse B16-F10–bearing skin of day 11–12 with isotype mAb or anti-CSF1R mAb treatment (representative of four mice for each group). Scale bar = 500 and 100 μm. **(B)** The ratio of CD169^+^ macrophages inside or outside of the tumor (left) in isotype mAb mice and the number of CD169^+^ macrophages in the tumor-bearing skin with treatment with isotype mAb or anti-CSF1R mAb (right) were analyzed using Imaris (*n* = 4 for each group, combined data from two experiments). P value was calculated using Student’s *t* test. **(C)** The number of intratumoral macrophages (CD169^−^ IBA1^+^ cells inside the tumor) with treatment with isotype mAb or anti-CSF1R mAb was analyzed using Imaris (*n* = 4 for each group, combined data from two experiments). P value was calculated using Student’s *t* test. **(D)** The numbers of LN SSM and CD169^+^ Mϕ after the two treatments of 200 μg isotype mAb or anti-CSF1R antibodies were subcutaneously injected into the mouse back skin were analyzed using flow cytometry (*n* = 3–5 for each group, data are representative of three independent experiments). P value was calculated using one-way ANOVA. **(E)** The tumor volumes of B16-F10 melanoma with isotype mAb in WT and B cell KO mice versus anti-CSF1R mAb in B cell KO mice (*n* = 15 for each group, data are combined from three independent experiments with four to five mice per group). Each circle represents one mouse. P value was calculated using one-way ANOVA.

We next determined the spatial organization of CD169^+^ macrophages in relation to the growing melanoma, which was localized in the adventitia, by confocal microscopy (Fig. 3 A). In mice treated with isotype control mAb, CD169^+^ IBA1^+^ macrophages were positioned around the edges and circumscribed the tumor margins, whereas CD169^−^ IBA1^+^ macrophages were observed infiltrating the tumor (Fig. 3 A). In contrast, the peri-tumoral CD169^+^ IBA1^+^ macrophages were depleted (Fig. 3 B), and tumors were larger in mice treated with anti-CSF1R mAb. It is of note that the number of intratumoral macrophages (CD169^−^ IBA1^+^ cells inside the tumor) was not significantly changed by anti-CSF1R mAb treatment (Fig. 3 C).

Not only skin CD169^+^ macrophages but also CD169^+^ F4/80^−^ SSM in the tdLN are CSF1R dependent (MacDonald et al., 2010; Grootveld et al., 2023; Dhenni et al., 2025), and it is therefore possible that the anti-CSF1R mAb exerted its effect via SSM in the tdLN (Pucci et al., 2016). To assess the contribution of SSM to the phenotype, we treated WT and B cell–deficient mice with anti-CSF1R mAb, since SSM are dependent on B cell–derived lymphotoxin signalling for their development and maintenance (Moseman et al., 2012; Phan et al., 2009). Indeed, B cell–deficient mice had fewer SSM in the tdLN (Fig. 3 D), and the number of CD169^+^ skin macrophages was unaffected by B cell deficiency but was depleted by CSF1R blockade (Fig. 3 D). Accordingly, CD169^+^ macrophage depletion in B cell–deficient mice also resulted in larger melanomas (Fig. 3 E). Thus, control of B16-F10 melanomas was achieved locally in the skin by B cell–independent CD169^+^ macrophages and not distally by B cell–dependent SSM in the tdLN.

### CD169^+^ skin macrophages control of tumors independent of T and B cells

**Figure S2.**
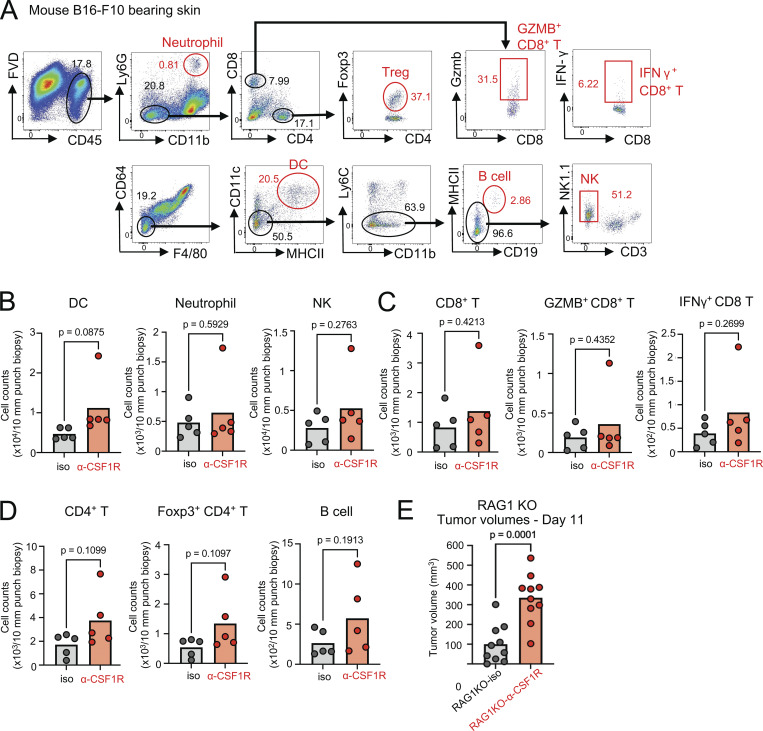
**CD169+ macrophages suppress tumour growth independent of acquired immunity** **. (A)** Gating strategy of immune cells in B16-F10–bearing skin (representative of five mice). **(B–D)** The numbers of DCs, neutrophils, natural killer (NK) cells, CD8^+^ T cells, cytotoxic CD8^+^ T cells, CD4^+^ T cells, Foxp3^+^ CD4^+^ T cells, and B cells in the tumors (day 7–11) with isotype mAb or anti-CSF1R mAb treatment (*n* = 5, data are representative of three independent experiments. Each circle represents one mouse). P value was calculated using Student’s *t* test. **(E)** The tumor volumes of B16-F10 melanoma with isotype mAb versus anti-CSF1R mAb in RAG1 KO mice (*n* = 10, data are combined from two independent experiments with five mice per group, each circle represents one mouse). P value was calculated using Student’s *t* test.

To determine the mechanism of tumor suppression by CD169^+^ macrophages, we first analyzed the tumor-infiltrating leukocytes by flow cytometry (Fig. S2 A). This showed that CSF1R blockade did not significantly alter the proportion of DCs, CD4^+^ T cells, CD8^+^ T cells, Foxp3^+^ CD4^+^ T cells, and B cells. There was no significant increase in the infiltration of CD8^+^ cytotoxic effector T cells expressing granzyme K and IFN-γ (Fig. S2, B–D). CD169^+^ macrophage depletion in recombinase-activating gene-1 (RAG1) knockout mice, which lack T and B cells, with anti-CSF1R mAb also led to unrestrained tumor growth (Fig. S2 E). Thus, containment of melanoma growth in this model is independent of T and B cells.

### CD169^+^ skin macrophages phagocytose live tumor cells

**Figure 4.**
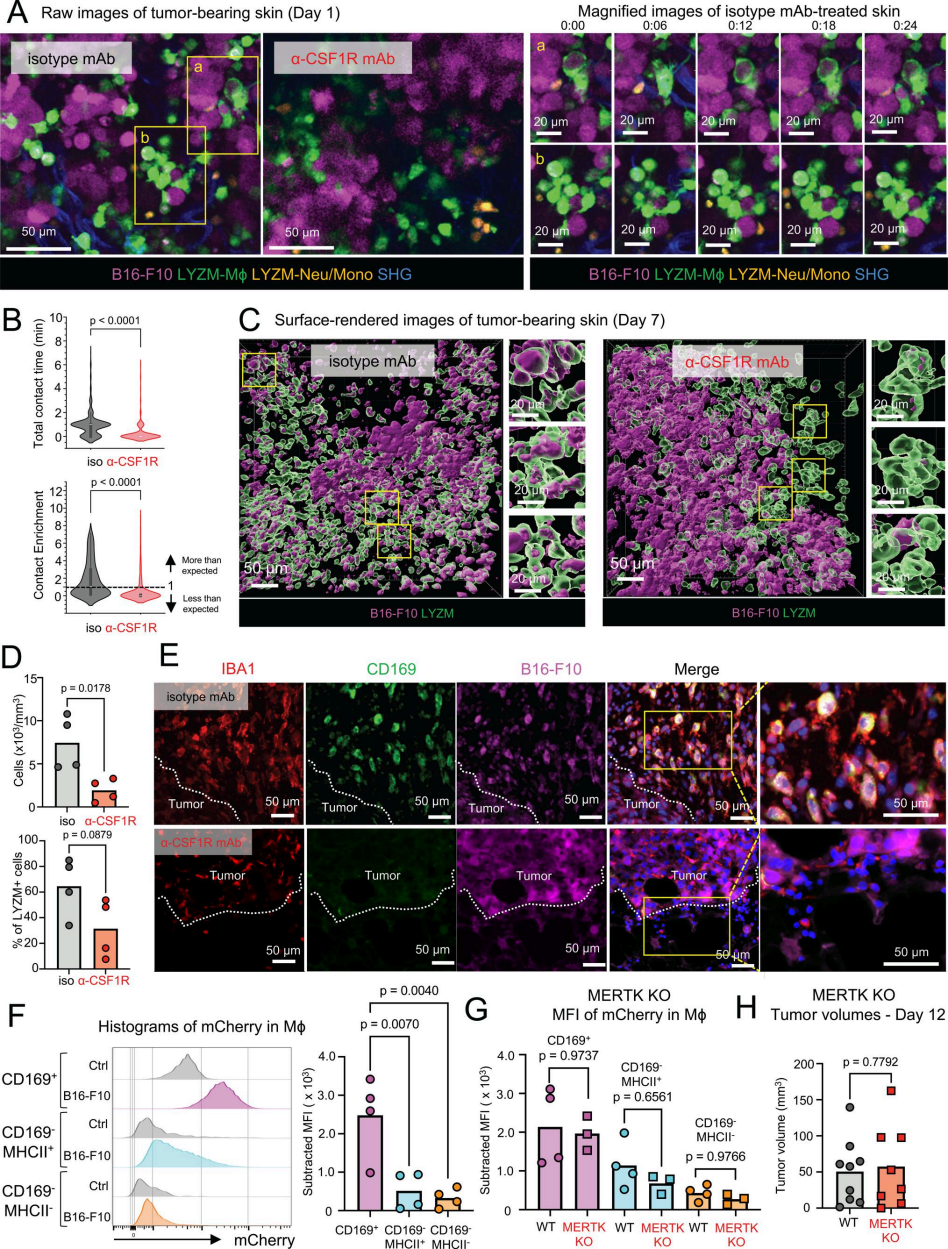
**Skin CD169^+^ macrophages ingest tumor cells. (A)** Direct interaction of LYZM^+^ cells and B16-F10 cells using intravital imaging on day 1. *Lysm*^Cre/+^.*Kikume*^LSL/LSL^ mice were subcutaneously treated with isotype mAb or anti-CSF1R mAb on days −3 and −1, and then mCherry-expressing B16-F10 cells were subcutaneously injected on day 0. Magnified images of the areas outlined in yellow rectangles (a and b) in isotype mAb-treated skin. Representative of four mice for each group. Scale bar = 50 and 20 μm. **(B)** Violin plots of total contact time (minutes) and contact enrichment (tumor cell–macrophage’s overlap fraction/the tumor cell occupancy) in the tumor-bearing skin with treatment of isotype mAb or anti-CSF1R mAb (day 1). P value was calculated using the Wilcoxon rank-sum test (representative of three mice for each group). **(C)** Engulfment of B16-F10 cells (mCherry^+^ cells) by LYZM^+^ cells using intravital imaging on day 7 (*n* = 4). Scale bar = 50 μm. **(D)** The number and percentages of mCherry-containing LYZM^+^ cells in the tumor-bearing skin with treatment of isotype mAb or anti-CSF1R mAb were quantified using Imaris (representative of four mice for each group, combined data of two experiments). P value was calculated using Student’s *t* test. **(E)** Immunostaining of IBA1 (red), CD169 (green), and DAPI (blue) in mouse mCherry-expressing (magenta) B16-F10–bearing skin on day 8 with treatment of isotype mAb or anti-CSF1R mAb (representative of four mice for each group). Scale bar = 50 μm. **(F)** Histograms of mCherry in CD169^+^ Mϕ, CD169^−^ MHCII^+^ Mϕ, and CD169^–^ MHCII^−^ Mϕ on day 8 of control (Ctrl) or mCherry-expressing B16-F10–bearing skin (B16-F10). MFI of mCherry in each macrophage subset in B16-F10 skin was subtracted by that in Ctrl skin (*n* = 4 for each group, data are representative of two independent experiments). P value was calculated using one-way ANOVA. **(G)** Subtracted MFI of mCherry in CD169^+^ Mϕ, CD169^–^ MHCII^+^ Mϕ, and CD169^–^ MHCII^−^ Mϕ on day 8 of mCherry-expressing B16-F10–bearing skin in WT or MERTK KO mice (*n* = 3–4 for each group, data are representative of two independent experiments). P value was calculated using one-way ANOVA. **(H)** The tumor volumes of B16-F10 melanoma in WT or MERTK KO mice (*n* = 8–9, data are combined from two independent experiments with four to five mice per group). Each circle represents one mouse. P value was calculated using Student’s *t* test.

We next performed intravital microscopy (IVM) to visualize dynamic interactions between macrophages and melanoma cells. Amelanotic B16-F10 cells expressing the red fluorescent protein mCherry (Jain et al., 2021; Tikoo et al., 2021) were injected subcutaneously into *Lysm*^Cre/+^.*Kikume*^LSL/LSL^ mice in which neutrophils, monocytes, macrophages, DCs, and type II lung alveolar epithelial cells are labelled green (Shi et al., 2018). We used a machine-learning approach to distinguish large slow-moving macrophages from smaller and more motile neutrophils and monocytes (Video 1 and Fig. 4 A). In mice treated with isotype mAb, macrophages were active and observed engulfing tumor cells by “hugging” and “nibbling” at them as early as day 1 after injection. Some macrophages also contained phagocytosed mCherry^+^ cells (Fig. 4 A). In contrast, in anti-CSF1R mAb-treated mice, we did not observe similar macrophage-tumor interactions, nor did we detect mCherry-containing macrophages (Fig. 4 A). Indeed, both contact time and contact enrichment (normalized overlap fraction) of macrophage and melanoma cells were significantly decreased in anti-CSF1R mAb-treated mice (Fig. 4 B). By day 7, there was a significant decrease in the number of mCherry^+^ macrophages in the anti-CSF1R mAb compared with isotype mAb-treated mice (Video 2 and Fig. 4, C and D). The phagocytosis of melanoma cells was confirmed by IF microscopy, which showed accumulation of mCherry signal in CD169^+^ macrophages at the tumor margins (Fig. 4 E) and quantified by flow cytometry (Fig. 4 F). Efferocytosis refers to the phagocytosis of apoptotic cells by recognition of the “eat me” signal phosphatidylserine by macrophage receptors such as MERTK (Burstyn-Cohen and Fresia, 2023), which is highly expressed by CD169^+^ skin macrophages (Fig. 1 D). However, when we compared MERTK-deficient (Thai et al., 2023) with WT mice, we did not observe any decrease in B16-F10-mCherry uptake (Fig. 4 G) nor did we detect any corresponding increase in tumor growth (Fig. 4 H). These data suggest that CD169^+^ skin macrophages directly phagocytose live tumor cells independent of MERTK to suppress their growth.

**Video 1.** **Macrophages directly interact with melanoma cells. (A–D)** Time-lapse intravital imaging of B16-F10 melanoma on day 1, with treatment of isotype mAb (A and B) and anti-CSF1R mAb (C and D). B16-F10-mCherry (magenta), SHG (blue), LYZM-Kikume-Mϕ (green), and LYZM-Kikume-neutrophil/monocytes (orange).

**Video 2.** **Macrophages ingest melanoma cells. (A–F)** Raw and surface-rendered 3D images of B16-F10 melanoma on day 7, with treatment of isotype mAb (A–C) and anti-CSF1R mAb (D–F). B16-F10-mCherry (magenta), SHG (blue), and LYZM-Kikume (green).

### CD169^+^ macrophages engulf melanoma in human skin

**Figure 5.**
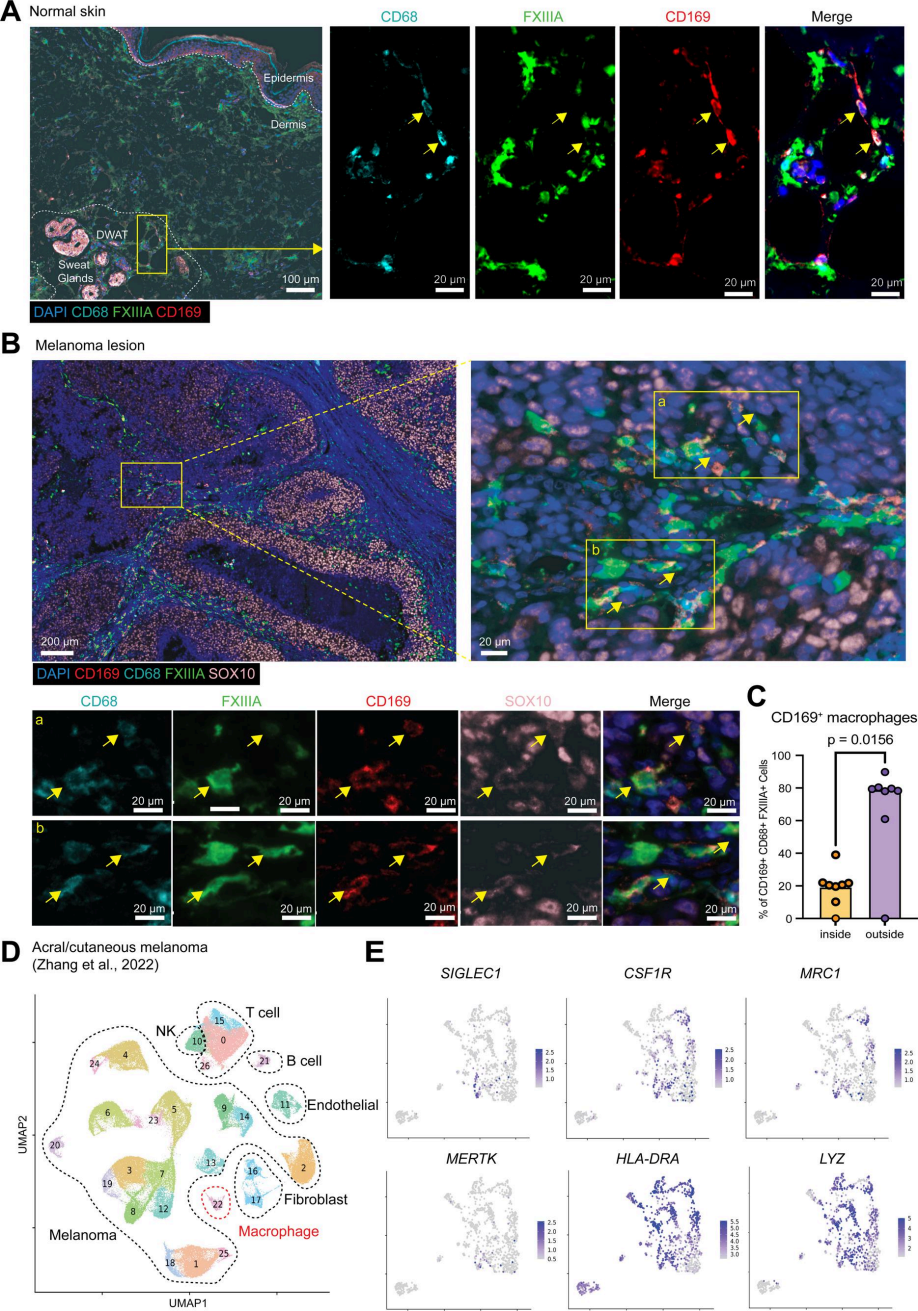
**CD169+ macrophages in human melanoma** **. (A)** Immunostaining of CD169^+^ macrophages in human normal/non-lesional skin with magnified images of the area outlined (yellow rectangle). Stained for DAPI (blue), CD68 (cyan), FXIIIA (green), and CD169 (red). CD169^+^ CD86^+^-positive cells were indicated with yellow arrows. Representative image of four samples. **(B)** Immunostaining of CD169^+^ macrophages in melanoma lesions with magnified images of the area outlined (yellow rectangles, a and b). Stained for DAPI (blue), CD68 (cyan), FXIIIA (green), CD169 (red), and SOX10 (pink). CD169^+^ CD86^+^-positive cells were indicated with yellow arrows. Representative image of eight samples. **(C)** The ratio of CD169^+^ macrophages (CD169^+^ CD68^+^ FXIIIA^+^ cells) inside or outside of primary melanoma lesions. Wilcoxon matched-pairs signed rank test was used. **(D)** UMAP plot illustrating immune cells and nonimmune cells in human melanoma lesions, color-coded for distinction. Cells were annotated based on the following gene expressions: melanoma cells (*MLANA*, *PMEL*, *MITF*, and *DCT*), fibroblasts (*COL1A1* and *COL3A1*), endothelial cells (*VWF* and *PECAM1*), T cells (*CD3D* and *CD3E*), B cells (*MS4A1* and *CD79A*), natural killer (NK) cells (*FGFBP2* and *KLRD1*), and macrophages (*CD68*, *CD14*, and *LYZ*). **(E)** Cells expressing *SIGLEC1*, *LYZ*, *MRC1*, *MERTK*, *HLA-DRA*, and *CSF1R* were plotted onto the UMAP of macrophages (cluster 22).

Finally, we showed that CD169^+^ macrophages were also present in healthy human skin, where they also resided in the DWAT, where they stained with CD68 but not the inflammatory marker FXIIIA (Fig. 5 A). We next analyzed CD169^+^ macrophages in human primary melanoma lesions. Immunohistochemical analysis revealed that, in seven out of eight patients with melanoma, CD169^+^ CD68^+^ macrophages expressing FXIIIA were observed among the palisading cells surrounding SOX10^+^ melanoma cells (Fig. 5, B and 5C). In some instances, these macrophages were closely associated with SOX10^+^ nuclear fragments. Thus, CD169^+^ macrophages may also reside in human skin, where they may also phagocytose melanoma cells. We reanalyzed a published dataset of single-cell RNA-sequencing (scRNA-seq) data of human acral and cutaneous melanoma lesions (Zhang et al., 2022), which annotated macrophages as CD68^+^ CD14^+^ LYZ^+^ cells (Fig. 5 D). We showed *SIGLEC1* (encoding CD169) was specifically expressed in a small subset of macrophages (Fig. 5 E). *SIGLEC1*^+^ macrophages also expressed *MRC1* (encoding CD206), *MERTK*, *HLA-DRA*, and *CSF1R* (Fig. 5 E), which is consistent with CD169 macrophages in mouse skin (Fig. 1 D).

The skin, as a barrier organ, contains various tissue-resident macrophages with specific functions dependent on their localization: LCs in the epidermis (Stingl et al., 1980), perivascular macrophages (CX3CR1^low^LYVE1^high^MHCII^low^) and perineural macrophages (CX3CR1^high^LYVE1^low^MHCII^high^) in the dermis (Chakarov et al., 2019; Kolter et al., 2019), and CCR2-LYVE1^+^ macrophages in the hypodermis (Voisin et al., 2023). Given that CD169^+^ macrophages express a different cell surface phenotype (CX3CR1^high^LYVE1^+^MHCII^mid^) and are located deep in the dermis and hypodermis, we believe they represent a distinct subset that may share some similarities with LYVE1^+^ hypodermal macrophages. CD169^+^ macrophages were originally described by Dupasquier et al. (2004). Etzerodt et al. showed that CD169^+^ macrophages infiltrate spontaneous Braf^V600E^-driven melanomas (Etzerodt et al., 2019). However, these cells were Ly6C negative. More recently, Park et al. showed that microbiota induced a type I IFN-dependent CD169^+^ skin macrophage subset in mice and humans that promoted the infiltration of γδ T cells to eliminate the bacteria *Staphylococcus aureus* (Park et al., 2022). Further, Li et al. demonstrated that CD169^+^ skin macrophages contribute to inflammation by promoting Th17 differentiation in an imiquimod-induced mouse psoriasis-like dermatitis model. Using the *Ms4a3*^CreERT2^.*Rosa26*^tdTomato^ lineage reporter mouse, they showed that the majority of CD169^+^ macrophages in their model were not derived from monocytes, suggesting that their numbers are locally maintained by self-renewing tissue-resident macrophages (Li et al., 2024). It was suggested that CD169^+^ macrophages comprise a distinct tissue-resident subset with pro-inflammatory functions in the skin.

We described the uptake of tumor cells by CD169^+^ macrophages in the periphery of B16-F10 melanoma. Phagocytosis of tumor cells by tissue-resident macrophages can limit tumor growth (Lecoultre et al., 2020). Liver-resident Kupffer cells can phagocytose primary and metastatic cancer cells and suppress tumor growth (Kimura et al., 2016; Deng et al., 2024). Brain-resident microglia are also capable of phagocytosis of tumor cells (Chen et al., 2023; Hutter et al., 2019). In the mouse B16-F10 melanoma model, inhibiting the expression of the “don’t eat me” signal CD47 significantly decreases tumor volumes (Wang et al., 2013). In addition, the presence of melanophages (skin macrophages that have ingested melanin) is associated with a better prognosis in human primary cutaneous melanoma (Handerson et al., 2007). These studies indicate the important role of phagocytosis in controlling primary melanoma lesions. In addition, macrophages can present ingested tumor antigens to CD4^+^ T cells via MHCII and promote adaptive anti-tumor immunity (Bogen et al., 2019). Some macrophages can also cross-present tumor antigens to activate CD8^+^ T cells (Asano et al., 2011; Modak et al., 2022). However, we have also shown that adaptive immunity was not essential for CD169^+^ macrophages to affect tumor growth. This may be due to the low immunogenicity of B16-F10 melanoma cells and the minimal role of adaptive immunity in tumor control in this model (Wang et al., 1998).

In conclusion, we have revealed a novel phagocytic tissue-resident macrophage subset in the skin that suppresses tumor growth in the mouse melanoma model. Importantly, we showed that analogous subpopulations of CD169^+^ macrophages are also present in normal human skin and in patients with melanoma, highlighting the therapeutic potential of targeting this specific subset.

## Materials and methods

### Mice

The Garvan Institute of Medical Research/St Vincent’s Hospital Animal Ethics Committee approved all animal experiments and procedures (#21_25, #24_34). All experiments were performed on age- and sex-matched 6- to 12-wk-old C57BL/6J genetic background mice housed under specific pathogen-free conditions. C57BL/6J mice and CD45.1 congenic mice (B6.SJL-Ptprca Pepcb/BoyJ) were obtained from Australian BioResources. Homozygous CD79a-ERT mice (B6.C-*Cd79a*^*tm3(cre/ERT2)Reth*^/EhobJ; RRID: IMSR_JAX:033026) were used as B cell–deficient mice. MERTK-deficient mice were previously described (Thai et al., 2023). Albino mice with spontaneous mutation in tyrosinase (B6(Cg)-*Tyr*^*c-2J*^/J; RRID: IMSR_JAX:000058) were crossed with *Lysm*^Cre^ (B6.129P2-*Lyz2*^*tm1(cre)Ifo*^/J; RRID: IMSR_JAX:004781) and *Kikume*^fl^ mice (RBRC04847; B6.Cg-Gt(ROSA)26Sor<tm1.1(CAG-kikGR)Kgwa>) (Tomura et al., 2014) to generate green fluorescent myeloid reporter mice for intravital imaging. *Ms4a3*^*Cre*^.*tdTom*^*LSL*^ mice were generated by crossing Ms4a3Cre knock-in mice (C57BL/6J-*Ms4a3*^*em2(cre)Fgnx*^/J; RPID: IMSR_JAX:036382) and LSL-tdTomato mice (B6.Cg-*Gt(ROSA)26Sor*^*tm14(CAG-tdTomato)Hze*^/J; RRID:IMSR_JAX:007914).

### B16-F10 melanoma model

Murine melanoma B16-F10 cells were purchased from the American Type Culture Collection. B16-F10-tyrosinase knockout and B16-F10-tyrosinase knockout-mCherry cells were generated as previously described (Jain et al., 2021; Tikoo et al., 2021). 1.0 × 10^5^ B16-F10 cells were injected intradermally or subcutaneously into the back skin.

### In vivo blocking of CSF1R

Anti-CSF1R (CD115) antibodies (AFS98) and rat IgG2a isotype control (2A3) were purchased from BioXcell. The mice were injected subcutaneously with 200 μg of anti-CSF1R antibodies or rat IgG2a isotype control antibodies on days −3 and −1 before intradermal/subcutaneous tumor injection.

### Single-cell preparation of tumor-bearing skin and LNs

The normal and tumor-bearing skin was resected using a 10-mm diameter punch biopsy (Acuderma), and inguinal LN was collected from each mouse. The tissues were incubated at 37°C in RPMI (Invitrogen) containing 2 mg/ml Collagenase D (Roche) and 10 μg/ml DNase I (Sigma-Aldrich) for 60 min for skin and 30 min for inguinal LNs. For intracellular staining of cytokines, ear pinnae were incubated in a digestion solution with 10 μg/ml of brefeldin A (Sigma-Aldrich). The digested skin and LNs were homogenized and filtered using a 70-μm cell strainer (BD Biosciences) to obtain single-cell suspensions.

### Flow cytometry

Single-cell suspensions were blocked with an anti-CD16/32 antibody (2.4G2; BD Biosciences) to prevent nonspecific antibody binding to Fc receptors. Cells were stained with the following antibodies: anti-mouse antibody to CD45 (30-F11; BD Biosciences), CD45.1 (A20; BioLegend), CD45.2 (104; BD Biosciences), Ly-6G (1A8; BD Biosciences), CD11b (M1/70; Invitrogen), CD11c (N418; BioLegend), EpCAM-1 (G8.8, eBioscience), F4/80 (BM8; BioLegend), CD64 (X54-5/7.1; BD Biosciences), MHC Class II (M5/114.152; Invitrogen), CD169 (SER-4; UCSF Hybridoma Core), CXCR3 (SA011F11; BioLegend), Ly6C (AL-21; BD Biosciences), MERTK (2B10C42; BioLegend), CD206 (C068C2; BioLegend), Lyve-1 (223322; R&D Systems), CX3CR1 (SA011F11; BioLegend), CD19 (1D3; BD Biosciences), CD4 (RM4-5; BioLegend), CD8b (YTS156.7.7; BioLegend), Foxp3 (FJK-16s; eBioscience), IFN-γ (XMG1.2; BioLegend), granzyme B (GB11; BD Biosciences), NK1.1 (PK136; BD Biosciences), and CD3e (500A2; eBioscience). FVD eFluor780 (Thermo Fisher Scientific) and/or DAPI were used to gate out dead cells. Cells were fixed with 0.2% formaldehyde (Sigma-Aldrich) or Foxp3/Transcription Factor Staining Buffer Set (Invitrogen) for intracellular cytokines and Foxp3 staining. Flow cytometry was performed using an LSRFortessa cell analyzer (BD Biosciences) and analyzed with FlowJo (TreeStar). Cell numbers of each cell subset were evaluated using Flow-Count Fluorospheres (Beckman Coulter) and presented as numbers per tissue after excluding doublet and dead cells.

### Immunostaining of tumor-bearing skin and LN

Mouse normal and melanoma-bearing skin and inguinal LN were fixed in 4% paraformaldehyde and embedded in OCT compound (Sakura Finetek). 7–10-μm slices were treated with Image-iT FX signal enhancer (Invitrogen) and incubated with rabbit anti-human/mouse polyclonal IBA-1 antibody (Invitrogen), anti-mouse CD169 antibody (SER-4; UCSF Hybridoma Core) conjugated to Alexa Fluor 647 or Alexa Fluor 488 (Thermo Fisher Scientific), Alexa Fluor 647 anti-mouse CD31 antibody (MEC13.3; BioLegend), or Alexa Fluor 647–conjugated anti-mouse LYVE-1 antibody (223322; R&D Systems) overnight at 4°C. Alexa Fluor 647 anti-goat IgG antibody (Invitrogen) was used for the second antibody. The slices were mounted with Fluoromount-G Mounting Medium (Thermo Fisher Scientific). Fluorescent images were analyzed with Leica DM5500 and Leica DMi6000 Inverted Microscopes.

### Irradiation BM chimeric mice

Recipient CD45.2 WT mice were lethally irradiated with 950 rads (9.5 Gy) using an X-ray irradiation device and received 5 × 10^6^ BM cells from congenic CD45.1 mice intravenously by retro-orbital injection. The chimeric mice were subjected to the indicated experiments 10–12 wk after reconstitution.

### Intravital imaging of tumor using two-photon microscopy

General anesthesia was induced with 100 mg/kg ketamine/20 mg/kg xylazine and maintained with 0.5–1.5% isoflurane supplemented with 100% oxygen at a flow rate of 500 ml/min via a nose cone. The anesthetized mouse was kept warm on a customized heated Biotherm SmartStage (CryoLogic) set to 37°C, and Lacri-Lube was applied to the eyes. Hair was removed, and a skin flap was used to expose the tumor.

Imaging was performed on an Olympus FVMPE-RS two-photon microscope (Evident) powered by an InSight X3 (Spectra-Physics) with a tuning range of 680–1300 nm and MaiTai DeepSee eHP (Spectra-Physics) with a tuning range 690–1040 nm NIR lasers. Images were acquired at 940 and 1060 nm. Filter Sets for NDD include: SHG (460–500 nm), fluorescence green (520–560 nm), and fluorescence mCherry (575–645 nm). We also used the FV30-FRCY5 with red barrier filter (575–645 nm), 650 nm dichroic mirror, and Cy5 barrier filter (660–750 nm) for red and far-red detection. 44–100 μm z-stacks were typically acquired at 4-μm z-step intervals. Image stacks were acquired at 50–60-s intervals for 30-min movies for 30–40 cycles.

### Image analysis

For analysis of immunohistochemistry (IHC) of mouse back skin, raw (.lif) image files were imported into Imaris (Oxford Instruments). Epidermis, dermis, DWAT, panniculus carnosus, and adventitia were identified based on anatomical structure and autofluorescence. To count macrophages in each layer of skin, regions of interest (ROIs) were created manually using Imaris surface function. Double-positive cells (IBA1 and CD169) in each ROI were identified as IBA^+^ cells located within ≤5.0 μm of CD169^+^ cells.

For analysis of 3D images obtained by two-photon microscopy, raw (.oir) image files were imported into Imaris (Oxford Instruments). Raw (LSM) image files were imported into Imaris (Oxford Instruments). Movies were stabilized for rotational/translational drift using the Imaris Correct Drift function. Surfaces were created for each channel (LYZM and mCherry) using Imaris surface function. To count mCherry-containing LYZM^+^ cells, first, ROIs were created as surfaces of LYZM^+^ cells, and then mCherry^+^ cells outside of LYZM^+^ cells were masked using the masking properties of Imaris to count only mCherry^+^ cells inside of LYZM^+^ cells.

### Discriminating between neutrophils/monocytes from macrophages

Accurately distinguishing neutrophils/monocytes from macrophages in IVM images is a challenging task due to several factors. First, some neutrophils/monocytes might appear substantially smaller than macrophages, making them difficult to detect, particularly in dense tissue environments. Second, while neutrophils/monocytes are typically smaller and exhibit more rapid movement compared with macrophages, it is not uncommon to observe static neutrophils/monocytes or rapidly migrating macrophages, which undermines simple heuristics based on size and speed alone. To overcome these limitations, we leveraged more sophisticated morphological and motility features derived from cell tracks rather than relying solely on individual cell characteristics to distinguish neutrophils/monocytes and macrophages.

To reduce the computational complexity, we restricted our analysis to the most informative z-slice from each 4D video sequence. Initial cell segmentation was performed using the pre-trained “cyto2_cp3” cellpose model (Stringer et al., 2021). However, due to the densely packed cellular environment and the small, irregular morphology of many immune cells, the segmentation output was suboptimal. To enhance segmentation performance, we manually annotated a subset of cells based on the initial cellpose output using the AnyLabeling tool (https://github.com/vietanhdev/anylabeling) and fine-tuned the segmentation model using a sparse loss function, which ignores the unannotated pixels during training. This approach significantly improved segmentation quality, enabling more accurate detection of small and morphologically complex cells.

Following segmentation, we employed the trackpy library (Allan et al., 2021) to link detected cells across frames and generate cell trajectories. We retained tracks with a minimum length of five frames and manually annotated a subset of them as either “neutrophil” or “macrophage.” To obtain class-balanced annotations, tracks were sorted by average cell area, and annotation alternated between the largest and smallest remaining tracks. This yielded 283 macrophage and 246 neutrophil tracks.

From each annotated track, we extracted a 15-dimensional feature vector comprising morphological and motility descriptors, such as average area, displacement, duration, velocity, and straightness. These features were used to train a support vector machine classifier with an 80:20 training-to-validation split. The trained model achieved an accuracy of ∼84% on the validation set and was subsequently used to classify the remaining unannotated tracks. While the classifier correctly identified most cell tracks, a small proportion of misclassifications remained. These were manually corrected via a custom-built interactive tool that enabled visual inspection and manual adjustment of predicted cell track labels.

### Quantification of macrophage-tumor cell contact

To quantify spatial interactions between macrophages and tumor cells, we employed an occupancy-normalized enrichment approach that addresses the challenge of reliably segmenting individual tumor cells. The tumor cell channel was thresholded and morphologically processed to create a binary occupancy map representing regions occupied by tumor cells. For each tracked macrophage at each time point, the segmentation mask was dilated by three pixels to account for potential segmentation inaccuracies, and the overlap fraction was calculated as the fraction of the dilated macrophage area that overlapped with the tumor occupancy map. While a contact event was defined when this overlap fraction exceeded 10%, we also computed contact enrichment, a metric that quantifies the preferential localization of macrophages to tumor-rich regions after normalization for tumor area occupancy.

Contact enrichment was quantified at the trajectory level. For each macrophage, the mean overlap fraction across its entire track was calculated and normalized to the global tumor occupancy (the average fraction of image area occupied by tumor signal). Enrichment values >1 indicate that macrophages preferentially localize to tumor-occupied regions relative to their spatial availability. This occupancy-normalized framework enables robust comparison across experimental conditions with varying tumor coverage, as it captures the propensity for macrophage–tumor association independently of absolute contact duration or discrete contact events.

### Multiplex IHC and IF of human healthy and melanoma skin

Formalin-fixed and paraffin-embedded (FFPE) primary melanoma skin (*n* = 8) and healthy skin (*n* = 4) biopsy samples were retrieved from the Melanoma Institute Australia Biospecimen Bank. Samples were retrieved as either single sample FFPE blocks for Opal multiplex IHC (mIHC) or as part of a FFPE tissue microarray for multiplex sequential IF (seqIF) using the COMET platform (Lunaphore Technologies). 4-µm sections from each FFPE block were sectioned onto charged slides. FFPE sections were baked for 60 min at 60°C, then deparaffinized in xylenes (Sigma-Aldrich) for 10 min. They were subsequently rehydrated through a graded ethanol series (100%, 90%, 70%, and 50%; Sigma-Aldrich) for 5 min in each solution. Sections were then washed in TRIS-buffered saline with Tween-20 (TBST) (pH 7.5, 0.1 M Tris HCl, and 0.15 M NaCl solution containing 0.05% Tween-20) for 5 min. Antigen retrieval (AR) and subsequent staining and imaging steps were then performed differently for each staining system.

For Opal mIHC, sections were submerged in either AR6 buffer (pH 6, 10 mM sodium citrate solution containing 0.05% Tween 20) or AR9 buffer (pH 9, 10 mM Tris-EDTA buffer solution containing 0.05% Tween 20), depending on the antibody intended for staining. AR was performed in a pressure cooker for 20 min at 95°C. Sections were cooled to RT before being washed twice in TBST for 2 min. Sections were incubated with 3% hydrogen peroxide (Sigma-Aldrich) for 30 min at room temperature (RT). Sections were washed in TBST before incubating with avidin and biotin (Life Technologies) for 10 min each. Sections were then incubated consecutively with donkey serum (Life Technologies, 20% in DPBS) and Antibody Diluent/Blocking Buffer (Akoya Biosciences) for 45 min each at 37°C. Sections were incubated with a single purified primary antibody diluted in Antibody Diluent/Blocking Buffer for 35 min at RT. After washing in TBST, sections were incubated with the secondary HRP antibody, either Opal Polymer HRP Ms + Rb (Akoya Biosciences) for 30 min or MACH-3 Mouse Probe and MACH-3 Rabbit HRP (Biocare Medical) consecutively for 20 min each. After washing in TBST, slides were incubated with the corresponding Opal fluorophore diluted 1:100 in 1× Plus Amplification Solution (Akoya Biosciences) for 10 min, followed by another round of AR. The primary antibodies CD169 (HSn7D2, Novus Biologicals), CD68 (Kp-1, Cell Marque), FXIIIA (Polyclonal, Affinity Biologicals), SOX10 (BC34, Biocare Medical); secondary antibody; and Opal fluorophore incubation were repeated for each marker. After staining was complete, sections were counterstained with DAPI (Cell Signalling Technologies, diluted 1:1,000 in TBST) for 30 min and then mounted using ProLong Diamond Antifade Mountant (Invitrogen, Thermo Fisher Scientific). Multiple field of view (FOV) images (magnification, 4×, 20×, and 40×) were acquired using the Mantra Quantitative Pathology Workstation (Akoya Biosciences) with MantraSnap software (version 1.0.3, Akoya Biosciences). Images were spectrally unmixed using inForm tissue analysis software (version 2.4.2, Akoya Biosciences). Single-channel TIFF images were exported from inForm and imported into Fiji (version 1.54q) to produce representative images.

For seqIF staining, sections were submerged in AR9 buffer for AR performed in a decloaking chamber (Biocare Medical) for 60 min at 99.5°C. Sections were cooled to RT before being washed twice in TBST for 2 min. Sections were incubated with Antibody Diluent/Blocking Buffer for 45 min at 37°C. Sections, antibodies, and staining reagents were then loaded onto the COMET instrument following the manufacturer’s protocols for automatic cyclic staining and imaging (Rivest et al., 2023). Sections were iteratively stained with primary antibodies (panel as previously described) and secondary antibodies for imaging prior to elution for the next cycle of staining. The acquired images (magnification, 20×) were automatically stitched and aligned, and multichannel OME.TIFF images with background fluorescence removed were exported using HORIZON software (Lunaphore Technologies).

Single-channel TIFF (mIHC) and multichannel OME.TIFF (seqIF) images were imported into HALO software (version 3.6.4134, Indica Labs) to quantify cell populations in the tissue. An AI algorithm based on DAPI nuclear staining was used to detect all individual cells. Positivity thresholds for each cell marker were adjusted to optimize the detection of true positive staining. Cell phenotypes were defined based on cell marker expression, and images were analyzed. The resulting cell quantities were computed as proportion of total nucleated cells and cells per mm^2^. For the mIHC images, the values for each FOV image were treated as replicates and averaged to represent the quantity of immune cells per sample.

### scRNA-seq of human melanoma

Raw expression matrices (filtered_feature_bc_matrix.h5) were downloaded from the National Center for Biotechnology Information Gene Expression Omnibus (NCBI GEO) entry GSE215121. For each sample .h5 file, counts were converted to a Seurat object using Read10X_h5 (“min.cells = 3”, “min.features = 200”). Each sample was log-normalized with NormalizeData. Samples were then merged into a single object using merge(). The merged dataset was then processed with the following: FindVariableFeatures (selection.method = “vst,” nfeatures = 2000), ScaleData (default parameters), and RunPCA (npcs = 50). Nearest neighbor graph was then computed with FindNeighbors (reduction = “pca,” dims = 1:30), followed by Louvain clustering with FindClusters (resolution = 0.5). Low-dimensional visualization was generated by RunUMAP (reduction = pca, dims = 1:30). Gene expression patterns for selected marker genes were visualized using FeaturePlot. The above analyses were completed in R version 4.5.0 and Seurat version 5.4.0.

### Statistical analysis

The data are presented as mean. Statistical analyses were performed using GraphPad Prism (GraphPad Software). Unless indicated otherwise, a parametric Student *t* test, a one-way ANOVA test, or a two-way ANOVA was used to compare groups.

### Online supplemental material

Fig. S1 contains the flow cytometry gating strategy for myeloid cells in the mouse back skin, flow cytometry analysis of FVD and DAPI in skin macrophage subsets, IHC of CD169^+^ macrophages and lymphatic or blood vessels, and IHC of CD169^+^ macrophages after the treatment with isotype mAb or anti-CSF1R mAb. Fig. S2 provides the gating strategy and numbers of immune cells in the B16-F10–bearing skin and the tumor volumes of B16-F10 in RAG1KO mice. Video 1 shows direct interactions of macrophages and B16-F10 melanoma cells on day 1. Video 2 demonstrates the ingestion of B16-F10 melanoma cells by macrophages on day 7.

## Data Availability

Human melanoma scRNA-seq data are available from the NCBI GEO entry GSE215121, and the code is available at https://doi.org/10.5281/zenodo.18603305 (Kyaw et al., 2026). All the other data are present in the article and supplementary information.

## References

[bib1] Abtin, A., R. Jain, A.J. Mitchell, B. Roediger, A.J. Brzoska, S. Tikoo, Q. Cheng, L.G. Ng, L.L. Cavanagh, U.H. von Andrian, . 2014. Perivascular macrophages mediate neutrophil recruitment during bacterial skin infection. Nat. Immunol. 15:45–53. 10.1038/ni.2769 24270515 PMC4097073

[bib60] Allan, D.B., T. Caswell, N.C. Keim, C.M. van der Wel, and R.W. Verweij. 2021. Soft-Matter/Trackpy: Trackpy v0.5.0. Zenodo. 10.5281/zenodo.4682814

[bib3] Asano, K., A. Nabeyama, Y. Miyake, C.-H. Qiu, A. Kurita, M. Tomura, O. Kanagawa, S. Fujii, and M. Tanaka. 2011. CD169-Positive macrophages dominate antitumor immunity by crosspresenting dead cell-associated antigens. Immunity. 34:85–95. 10.1016/j.immuni.2010.12.011 21194983

[bib4] Barreiro, O., D. Cibrian, C. Clemente, D. Alvarez, V. Moreno, Í. Valiente, A. Bernad, D. Vestweber, A.G. Arroyo, P. Martín, . 2016. Pivotal role for skin transendothelial radio-resistant anti-inflammatory macrophages in tissue repair. Elife. 5:e15251. 10.7554/eLife.15251 27304075 PMC4961461

[bib5] Binnewies, M., E.W. Roberts, K. Kersten, V. Chan, D.F. Fearon, M. Merad, L.M. Coussens, D.I. Gabrilovich, S. Ostrand-Rosenberg, C.C. Hedrick, . 2018. Understanding the tumor immune microenvironment (TIME) for effective therapy. Nat. Med. 24:541–550. 10.1038/s41591-018-0014-x 29686425 PMC5998822

[bib6] Bogen, B., M. Fauskanger, O.A. Haabeth, and A. Tveita. 2019. CD4+ T cells indirectly kill tumor cells via induction of cytotoxic macrophages in mouse models. Cancer Immunol. Immunother. 68:1865–1873. 10.1007/s00262-019-02374-0 31448380 PMC11028095

[bib7] Burstyn-Cohen, T., and R. Fresia. 2023. TAM receptors in phagocytosis: Beyond the mere internalization of particles. Immunol. Rev. 319:7–26. 10.1111/imr.13267 37596991

[bib8] Carrasco, Y.R., and F.D. Batista. 2007. B cells acquire particulate antigen in a macrophage-rich area at the boundary between the follicle and the subcapsular sinus of the lymph node. Immunity. 27:160–171. 10.1016/j.immuni.2007.06.007 17658276

[bib9] Chakarov, S., H.Y. Lim, L. Tan, S.Y. Lim, P. See, J. Lum, X.-M. Zhang, S. Foo, S. Nakamizo, K. Duan, . 2019. Two distinct interstitial macrophage populations coexist across tissues in specific subtissular niches. Science. 363:eaau0964. 10.1126/science.aau0964 30872492

[bib10] Chen, D., S.K. Varanasi, T. Hara, K. Traina, M. Sun, B. McDonald, Y. Farsakoglu, J. Clanton, S. Xu, L. Garcia-Rivera, . 2023. CTLA-4 blockade induces a microglia-Th1 cell partnership that stimulates microglia phagocytosis and anti-tumor function in glioblastoma. Immunity. 56:2086–2104.e8. 10.1016/j.immuni.2023.07.015 37572655 PMC11800830

[bib11] DeNardo, D.G., and B. Ruffell. 2019. Macrophages as regulators of tumour immunity and immunotherapy. Nat. Rev. Immunol. 19:369–382. 10.1038/s41577-019-0127-6 30718830 PMC7339861

[bib12] Deng, Z., P.-L. Loyher, T. Lazarov, L. Li, Z. Shen, B. Bhinder, H. Yang, Y. Zhong, A. Alberdi, J. Massague, . 2024. The nuclear factor ID3 endows macrophages with a potent anti-tumour activity. Nature. 626:864–873. 10.1038/s41586-023-06950-4 38326607 PMC10881399

[bib13] Dhenni, R., A.C. Hoppé, A. Reynaldi, W. Kyaw, N.T. Handoko, A.K. Grootveld, Y.H. Keith, N.D. Bhattacharyya, H.I. Ahel, A.J. Telfser, . 2025. Macrophages direct location-dependent recall of B cell memory to vaccination. Cell. 188:3477–3496.e22. 10.1016/j.cell.2025.04.005 40300604

[bib14] Dupasquier, M., P. Stoitzner, A. van Oudenaren, N. Romani, and P.J.M. Leenen. 2004. Macrophages and dendritic cells constitute a major subpopulation of cells in the mouse dermis. J. Invest. Dermatol. 123:876–879. 10.1111/j.0022-202X.2004.23427.x 15482474

[bib15] Etzerodt, A., K. Tsalkitzi, M. Maniecki, W. Damsky, M. Delfini, E. Baudoin, M. Moulin, M. Bosenberg, J.H. Graversen, N. Auphan-Anezin, . 2019. Specific targeting of CD163+ TAMs mobilizes inflammatory monocytes and promotes T cell-mediated tumor regression. J. Exp. Med. 216:2394–2411. 10.1084/jem.20182124 31375534 PMC6781002

[bib16] Grabert, K., A. Sehgal, K.M. Irvine, E. Wollscheid-Lengeling, D.D. Ozdemir, J. Stables, G.A. Luke, M.D. Ryan, A. Adamson, N.E. Humphreys, . 2020. A transgenic line that reports CSF1R protein expression provides a definitive marker for the mouse mononuclear phagocyte system. J. Immunol. 205:3154–3166. 10.4049/jimmunol.2000835 33139489

[bib17] Grootveld, A.K., W. Kyaw, V. Panova, A.W.Y. Lau, E. Ashwin, G. Seuzaret, R. Dhenni, N.D. Bhattacharyya, W.H. Khoo, M. Biro, . 2023. Apoptotic cell fragments locally activate tingible body macrophages in the germinal center. Cell. 186:1144–1161.e18. 10.1016/j.cell.2023.02.004 36868219 PMC7614509

[bib18] Habib, S., G. Osborn, Z. Willsmore, M.W. Chew, S. Jakubow, A. Fitzpatrick, Y. Wu, K. Sinha, H. Lloyd-Hughes, J.L.C. Geh, . 2024. Tumor associated macrophages as key contributors and targets in current and future therapies for melanoma. Expert Rev. Clin. Immunol. 20:895–911. 10.1080/1744666X.2024.2326626 38533720 PMC11286214

[bib19] Handerson, T., A. Berger, M. Harigopol, D. Rimm, C. Nishigori, M. Ueda, E. Miyoshi, N. Taniguchi, and J. Pawelek. 2007. Melanophages reside in hypermelanotic, aberrantly glycosylated tumor areas and predict improved outcome in primary cutaneous malignant melanoma. J. Cutan. Pathol. 34:679–686. 10.1111/j.1600-0560.2006.00681.x 17696914

[bib20] Hume, D.A., and K.P.A. MacDonald. 2012. Therapeutic applications of macrophage colony-stimulating factor-1 (CSF-1) and antagonists of CSF-1 receptor (CSF-1R) signaling. Blood. 119:1810–1820. 10.1182/blood-2011-09-379214 22186992

[bib21] Hutter, G., J. Theruvath, C.M. Graef, M. Zhang, M.K. Schoen, E.M. Manz, M.L. Bennett, A. Olson, T.D. Azad, R. Sinha, . 2019. Microglia are effector cells of CD47-SIRPα antiphagocytic axis disruption against glioblastoma. Proc. Natl. Acad. Sci. USA. 116:997–1006. 10.1073/pnas.1721434116 30602457 PMC6338872

[bib22] Jain, R., S. Tikoo, K. On, B. Martinez, S. Dervish, L.L. Cavanagh, and W. Weninger. 2021. Visualizing murine breast and melanoma tumor microenvironment using intravital multiphoton microscopy. STAR Protoc. 2:100722. 10.1016/j.xpro.2021.100722 34458865 PMC8379651

[bib23] Jensen, T.O., H. Schmidt, H.J. Møller, M. Høyer, M.B. Maniecki, P. Sjoegren, I.J. Christensen, and T. Steiniche. 2009. Macrophage markers in serum and tumor have prognostic impact in American joint committee on cancer stage I/II melanoma. J Clin. Oncol. 27:3330–3337. 10.1200/JCO.2008.19.9919 19528371

[bib24] Junt, T., E.A. Moseman, M. Iannacone, S. Massberg, P.A. Lang, M. Boes, K. Fink, S.E. Henrickson, D.M. Shayakhmetov, N.C. Di Paolo, . 2007. Subcapsular sinus macrophages in lymph nodes clear lymph-borne viruses and present them to antiviral B cells. Nature. 450:110–114. 10.1038/nature06287 17934446

[bib25] Kabashima, K., T. Honda, F. Ginhoux, and G. Egawa. 2019. The immunological anatomy of the skin. Nat. Rev. Immunol. 19:19–30. 10.1038/s41577-018-0084-5 30429578

[bib26] Kimura, Y., A. Inoue, S. Hangai, S. Saijo, H. Negishi, J. Nishio, S. Yamasaki, Y. Iwakura, H. Yanai, and T. Taniguchi. 2016. The innate immune receptor Dectin-2 mediates the phagocytosis of cancer cells by Kupffer cells for the suppression of liver metastasis. Proc. Natl. Acad. Sci. USA. 113:14097–14102. 10.1073/pnas.1617903113 27872290 PMC5150405

[bib27] Kolter, J., R. Feuerstein, P. Zeis, N. Hagemeyer, N. Paterson, P. d’Errico, S. Baasch, L. Amann, T. Masuda, A. Lösslein, . 2019. A subset of skin macrophages contributes to the surveillance and regeneration of local nerves. Immunity. 50:1482–1497.e7. 10.1016/j.immuni.2019.05.009 31201094

[bib28] Komohara, Y., K. Ohnishi, and M. Takeya. 2017. Possible functions of CD169-positive sinus macrophages in lymph nodes in anti-tumor immune responses. Cancer Sci. 108:290–295. 10.1111/cas.13137 28002629 PMC5378284

[bib29] Kyaw, W., Y. Keith, and T. Phan. 2026. Seurat Analysis for Reprocessing PMID: 36433984 for Keith et al. Version v1. Zenodo. 10.5281/zenodo.18603305

[bib30] Lecoultre, M., V. Dutoit, and P.R. Walker. 2020. Phagocytic function of tumor-associated macrophages as a key determinant of tumor progression control: A review. J. Immunother. Cancer. 8:e001408. 10.1136/jitc-2020-001408 33335026 PMC7747550

[bib31] Li, M., W. Yu, Z. Liu, and S. Liu. 2024. CD169+ skin macrophages function as a specialized subpopulation in promoting psoriasis-like skin disease in mice. Int. J. Mol. Sci. 25:5705. 10.3390/ijms25115705 38891893 PMC11171985

[bib33] Liu, Z., Y. Gu, S. Chakarov, C. Bleriot, I. Kwok, X. Chen, A. Shin, W. Huang, R.J. Dress, C.-A. Dutertre, . 2019. Fate mapping via Ms4a3-expression history traces monocyte-derived cells. Cell. 178:1509–1525.e19. 10.1016/j.cell.2019.08.009 31491389

[bib34] MacDonald, K.P.A., J.S. Palmer, S. Cronau, E. Seppanen, S. Olver, N.C. Raffelt, R. Kuns, A.R. Pettit, A. Clouston, B. Wainwright, . 2010. An antibody against the colony-stimulating factor 1 receptor depletes the resident subset of monocytes and tissue- and tumor-associated macrophages but does not inhibit inflammation. Blood. 116:3955–3963. 10.1182/blood-2010-02-266296 20682855

[bib35] Malissen, B., S. Tamoutounour, and S. Henri. 2014. The origins and functions of dendritic cells and macrophages in the skin. Nat. Rev. Immunol. 14:417–428. 10.1038/nri3683 24854591

[bib36] Mass, E., F. Nimmerjahn, K. Kierdorf, and A. Schlitzer. 2023. Tissue-specific macrophages: How they develop and choreograph tissue biology. Nat. Rev. Immunol. 23:563–579. 10.1038/s41577-023-00848-y 36922638 PMC10017071

[bib37] Modak, M., A.-K. Mattes, D. Reiss, W. Skronska-Wasek, R. Langlois, N. Sabarth, R. Konopitzky, F. Ramirez, K. Lehr, T. Mayr, . 2022. CD206+ tumor-associated macrophages cross-present tumor antigen and drive antitumor immunity. JCI Insight. 7:e155022. 10.1172/jci.insight.155022 35503656 PMC9220841

[bib38] Moran, I., A.K. Grootveld, A. Nguyen, and T.G. Phan. 2019. Subcapsular sinus macrophages: The seat of innate and adaptive memory in murine lymph nodes. Trends Immunol. 40:35–48. 10.1016/j.it.2018.11.004 30502023

[bib39] Moran, I., A. Nguyen, W.H. Khoo, D. Butt, K. Bourne, C. Young, J.R. Hermes, M. Biro, G. Gracie, C.S. Ma, . 2018. Memory B cells are reactivated in subcapsular proliferative foci of lymph nodes. Nat. Commun. 9:3372. 10.1038/s41467-018-05772-7 30135429 PMC6105623

[bib40] Moseman, E.A., M. Iannacone, L. Bosurgi, E. Tonti, N. Chevrier, A. Tumanov, Y.-X. Fu, N. Hacohen, and U.H. von Andrian. 2012. B cell maintenance of subcapsular sinus macrophages protects against a fatal viral infection independent of adaptive immunity. Immunity. 36:415–426. 10.1016/j.immuni.2012.01.013 22386268 PMC3359130

[bib41] Park, Y.J., B.H. Kang, H.-J. Kim, J.E. Oh, and H.K. Lee. 2022. A microbiota-dependent subset of skin macrophages protects against cutaneous bacterial infection. Front. Immunol. 13:799598. 10.3389/fimmu.2022.799598 35757750 PMC9218056

[bib42] Phan, T.G., J.A. Green, E.E. Gray, Y. Xu, and J.G. Cyster. 2009. Immune complex relay by subcapsular sinus macrophages and noncognate B cells drives antibody affinity maturation. Nat. Immunol. 10:786–793. 10.1038/ni.1745 19503106 PMC2776777

[bib43] Phan, T.G., I. Grigorova, T. Okada, and J.G. Cyster. 2007. Subcapsular encounter and complement-dependent transport of immune complexes by lymph node B cells. Nat. Immunol. 8:992–1000. 10.1038/ni1494 17660822

[bib44] Phan, T.G., K.N. Weilbaecher, R. Aft, P.I. Croucher, and C.L. Chaffer. 2024. Chemotherapy and the extra-tumor immune microenvironment: EXTRA-TIME. Cancer Discov. 14:643–647. 10.1158/2159-8290.CD-23-1543 38571433 PMC12239161

[bib45] Pucci, F., C. Garris, C.P. Lai, A. Newton, C. Pfirschke, C. Engblom, D. Alvarez, M. Sprachman, C. Evavold, A. Magnuson, . 2016. SCS macrophages suppress melanoma by restricting tumor-derived vesicle-B cell interactions. Science. 352:242–246. 10.1126/science.aaf1328 26989197 PMC4960636

[bib46] Rivest, F., D. Eroglu, B. Pelz, J. Kowal, A. Kehren, V. Navikas, M.G. Procopio, P. Bordignon, E. Pérès, M. Ammann, . 2023. Fully automated sequential immunofluorescence (seqIF) for hyperplex spatial proteomics. Sci. Rep. 13:16994. 10.1038/s41598-023-43435-w 37813886 PMC10562446

[bib47] Sasaki, Y., K. Ohsawa, H. Kanazawa, S. Kohsaka, and Y. Imai. 2001. Iba1 is an actin-cross-linking protein in macrophages/microglia. Biochem. Biophysical Res. Commun. 286:292–297. 10.1006/bbrc.2001.5388 11500035

[bib48] Shi, J., L. Hua, D. Harmer, P. Li, and G. Ren. 2018. Cre driver mice targeting macrophages. Methods Mol. Biol. 1784:263–275. 10.1007/978-1-4939-7837-3_24 29761406 PMC6331202

[bib49] Stingl, G., K. Tamaki, and S.I. Katz. 1980. Origin and function of epidermal Langerhans cells. Immunol. Rev. 53:149–174. 10.1111/j.1600-065X.1980.tb01043.x 6162777

[bib50] Stringer, C., T. Wang, M. Michaelos, and M. Pachitariu. 2021. Cellpose: A generalist algorithm for cellular segmentation. Nat. Methods. 18:100–106. 10.1038/s41592-020-01018-x 33318659

[bib51] Suan, D., A. Nguyen, I. Moran, K. Bourne, J.R. Hermes, M. Arshi, H.R. Hampton, M. Tomura, Y. Miwa, A.D. Kelleher, . 2015. T follicular helper cells have distinct modes of migration and molecular signatures in naive and memory immune responses. Immunity. 42:704–718. 10.1016/j.immuni.2015.03.002 25840682

[bib52] Thai, L.M., L. O’Reilly, S. Reibe-Pal, N. Sue, H. Holliday, L. Small, C. Schmitz-Peiffer, R. Dhenni, V. Wang-Wei Tsai, N. Norris, . 2023. β-cell function is regulated by metabolic and epigenetic programming of islet-associated macrophages, involving Axl, Mertk, and TGFβ receptor signaling. iScience. 26:106477. 10.1016/j.isci.2023.106477 37091234 PMC10113792

[bib53] Tikoo, S., R. Jain, F. Tomasetig, K. On, B. Martinez, C. Heu, D. Stehle, P. Obeidy, D. Guo, J.N. Vincent, . 2021. Amelanotic B16-F10 melanoma compatible with advanced three-dimensional imaging modalities. J. Invest. Dermatol. 141:2090–2094.e6. 10.1016/j.jid.2021.01.025 33675788

[bib54] Tomura, M., A. Hata, S. Matsuoka, F.H.W. Shand, Y. Nakanishi, R. Ikebuchi, S. Ueha, H. Tsutsui, K. Inaba, K. Matsushima, . 2014. Tracking and quantification of dendritic cell migration and antigen trafficking between the skin and lymph nodes. Sci. Rep. 4:6030. 10.1038/srep06030 25112380 PMC4129424

[bib55] Torisu, H., M. Ono, H. Kiryu, M. Furue, Y. Ohmoto, J. Nakayama, Y. Nishioka, S. Sone, and M. Kuwano. 2000. Macrophage infiltration correlates with tumor stage and angiogenesis in human malignant melanoma: Possible involvement of TNFalpha and IL-1alpha. Int. J. Cancer 85:182–188. 10.1002/(SICI)1097-0215(20000115)85:2<182::AID-IJC6>3.0.CO;2 10629075

[bib56] Voisin, B., V. Nadella, T. Doebel, S. Goel, K. Sakamoto, O. Ayush, J.-H. Jo, M.C. Kelly, T. Kobayashi, J.X. Jiang, . 2023. Macrophage-mediated extracellular matrix remodeling controls host Staphylococcus aureus susceptibility in the skin. Immunity. 56:1561–1577.e9. 10.1016/j.immuni.2023.06.006 37402364 PMC10467568

[bib57] Wang, J., S. Saffold, X. Cao, J. Krauss, and W. Chen. 1998. Eliciting T cell immunity against poorly immunogenic tumors by immunization with dendritic cell-tumor fusion vaccines. J. Immunol. 161:5516–5524. 10.4049/jimmunol.161.10.5516 9820528

[bib58] Wang, Y., Z. Xu, S. Guo, L. Zhang, A. Sharma, G.P. Robertson, and L. Huang. 2013. Intravenous delivery of siRNA targeting CD47 effectively inhibits melanoma tumor growth and lung metastasis. Mol. Ther. 21:1919–1929. 10.1038/mt.2013.135 23774794 PMC3808133

[bib59] Zhang, C., H. Shen, T. Yang, T. Li, X. Liu, J. Wang, Z. Liao, J. Wei, J. Lu, H. Liu, . 2022. A single-cell analysis reveals tumor heterogeneity and immune environment of acral melanoma. Nat. Commun. 13:7250. 10.1038/s41467-022-34877-3 36433984 PMC9700682

